# A Novel Type of Nutritional Ant–Plant Interaction: Ant Partners of Carnivorous Pitcher Plants Prevent Nutrient Export by Dipteran Pitcher Infauna

**DOI:** 10.1371/journal.pone.0063556

**Published:** 2013-05-22

**Authors:** Mathias Scharmann, Daniel G. Thornham, T. Ulmar Grafe, Walter Federle

**Affiliations:** 1 Department of Animal Ecology and Tropical Biology, Biocenter, University of Würzburg, Würzburg, Germany; 2 Department of Zoology, University of Cambridge, Cambridge, United Kingdom; 3 Division of Biology, University of Glamorgan, Pontypridd, United Kingdom; 4 Department of Biology, Universiti Brunei Darussalam, Gadong, Brunei Darussalam; Centro de Investigación y de Estudios Avanzados, Mexico

## Abstract

Many plants combat herbivore and pathogen attack indirectly by attracting predators of their herbivores. Here we describe a novel type of insect–plant interaction where a carnivorous plant uses such an indirect defence to prevent nutrient loss to kleptoparasites. The ant *Camponotus schmitzi* is an obligate inhabitant of the carnivorous pitcher plant *Nepenthes bicalcarata* in Borneo. It has recently been suggested that this ant–plant interaction is a nutritional mutualism, but the detailed mechanisms and the origin of the ant-derived nutrient supply have remained unexplained. We confirm that *N. bicalcarata* host plant leaves naturally have an elevated ^15^N/^14^N stable isotope abundance ratio (δ^15^N) when colonised by *C. schmitzi*. This indicates that a higher proportion of the plants’ nitrogen is insect-derived when *C. schmitzi* ants are present (ca. 100%, vs. 77% in uncolonised plants) and that more nitrogen is available to them. We demonstrated direct flux of nutrients from the ants to the host plant in a ^15^N pulse-chase experiment. As *C. schmitzi* ants only feed on nectar and pitcher contents of their host, the elevated foliar δ^15^N cannot be explained by classic ant-feeding (myrmecotrophy) but must originate from a higher efficiency of the pitcher traps. We discovered that *C. schmitzi* ants not only increase the pitchers' capture efficiency by keeping the pitchers’ trapping surfaces clean, but they also reduce nutrient loss from the pitchers by predating dipteran pitcher inhabitants (infauna). Consequently, nutrients the pitchers would have otherwise lost *via* emerging flies become available as ant colony waste. The plants’ prey is therefore conserved by the ants. The interaction between *C. schmitzi*, *N. bicalcarata* and dipteran pitcher infauna represents a new type of mutualism where animals mitigate the damage by nutrient thieves to a plant.

## Introduction

**Figure 1:**
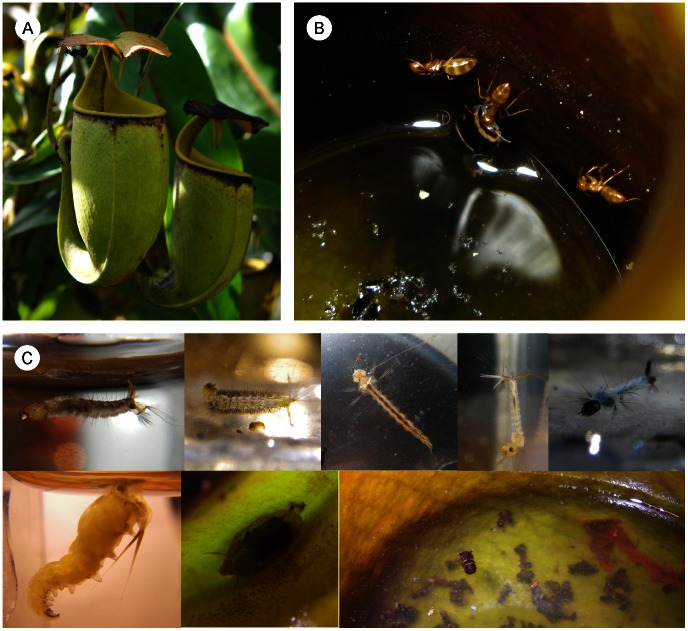
Association between *Nepenthes bicalcarata* pitcher plants, *Camponotus schmitzi* ants, and fly larvae that develop in the pitchers. A. Pair of *N. bicalcarata* upper pitchers. B. *C. schmitzi* workers retrieve a drowned cockroach from the fluid inside a pitcher. C. examples of the rich dipteran infauna of *N. bicalcarata*; from left to right; top row: *Toxorhynchites* sp., *Tripteroides* sp., *Culex* morphospecies 1, *Culex* morphospecies 2, *Uranotaenia* sp.; bottom row: *Wilhelmina nepenthicola*, large puparium (cf. Phoridae) hanging under the pitcher rim, large culicid pupa at fluid surface (other pupae, culicid larvae and *Polypedilum* (Chironomidae) larvae living in protective cases are also visible).

Insects and flowering plants are the two most diverse lineages of eukaryotes today, and their manifold interactions affect virtually all terrestrial life. Both insect and plant diversity can be explained to some extent by their ability to form complex interactions with each other. Relationships between ants and plants are particularly widespread because of the ants’ abundance and the mutual benefit for both partners [1], [2]. Ants benefit plants in several ways, e.g. by defending them against herbivory or preventing them from being overgrown by vines. Ants are lured by food rewards to the plant, where they attack herbivorous insects or remove their eggs from the plant. Tropical ant-plants (myrmecophytes) even offer special cavities (domatia) as nesting space for ants to ensure their permanent presence on the plant. Carnivorous plants are well-known exceptions to this pattern of apparent harmony, often killing visiting ants to use them as fertiliser in nutrient-poor habitats. Nevertheless, a unique ant–plant interaction from the peat swamp forests of Borneo combines the seemingly incompatible realms of carnivory and myrmecophytism. The pitcher plant *Nepenthes bicalcarata* Hook. f. (Fig. 1 A) traps and digests almost any insect, and ants in particular [3]–[5], yet simultaneously houses an obligate ant partner, *Camponotus schmitzi* Stärcke [6]–[8](Fig. 1 B). *Nepenthes* are tropical perennials whose insect traps are jug-shaped structures at the tips of their leaves, filled with liquid. The plants undergo an ontogenetic shift in growth habit, starting off as rosettes, bearing “lower” or “ground” pitchers, and developing into a liana producing “upper“ or “aerial“ pitchers [9], [10].

*C. schmitzi* ants live exclusively on *N. bicalcarata*, where they rear their brood in the hollow pitcher tendrils. They are able to forage unharmed in the pitchers because of their unique ability to walk across the slippery trapping surfaces on the pitcher rim [11], and to swim and dive in the digestive fluid [8], [12]. This allows the ants to exploit the pitcher as a food resource, removing and consuming prey captured by their host plant, as well as harvesting extrafloral nectar produced at the slippery pitcher rim [8], [13].

Since *Nepenthes* pitcher plants grow more slowly when deprived of prey [14], a competitive situation between host and ants might arise, which has led some authors to conclude that *C. schmitzi* ants are parasites [15]. However, the swollen, hollow and lignified pitcher tendrils of *N. bicalcarata* are unique in the genus, and it is likely that this trait is an adaptation to colonisation by the partner ants.

The apparent paradox that *N. bicalcarata* houses ants that “steal” the prey has inspired several studies testing the possible benefits of *C. schmitzi* to *N. bicalcarata* [4], [5], [8], [16]. Except for one investigation of herbivore defence [16], all of them concern mechanisms resulting in an enhanced nutrient acquisition from prey insects mediated by the ant. A recent study showed that the fitness of *N. bicalcarata* correlates with ant occupation [17]. However, as the ants might prefer to colonise larger and faster-growing plants that hence offer more food and nesting space, it remains unclear whether the presence of *C. schmitzi* is a cause or a consequence of the plant's higher fitness. Moreover, a flux of nutrients from the ants to the host plant (myrmecotrophy) has still not been demonstrated.

Bazile et al. [17] reported that *C. schmitzi*-inhabited *N. bicalcarata* plants were more enriched in ^15^N and concluded that the association is a nutritional mutualism, in which host plants derive large amounts of their foliar nitrogen from ant wastes. However, the detailed mechanism of this nutrition is unclear. Evidently, if *C. schmitzi* ants feed only on nectar provided by the host plant and on animal prey captured by the pitchers, a net gain of nutrients for *N. bicalcarata* would be impossible. Nutrient gain in a myrmecotrophic relationship can only occur if the ants acquire nutrients from sources unavailable to the host plant itself. *C. schmitzi* ants have a cryptic lifestyle, and consistent with previous reports [8], [16], [18], we have only very rarely observed them outside *N. bicalcarata* (during at total of >200 hours of day and night-time observations, we saw them only twice walking on the leaves of another plant that was in direct contact with *N. bicalcarata*). Thus, if these ants are indeed nutritional mutualists, how can they increase the nutrient supply for *N. bicalcarata?*

It is possible that the predatory activity of *C. schmitzi* within the pitchers [8], [16] helps nutrient retention: *Nepenthes* pitchers are not only traps but also act as phytotelmata, providing habitat for many different organisms that exploit the abundance of dead organic material [19]. *N. bicalcarata* pitchers have been found to contain an exceptionally rich dipteran infauna (14 taxa, examples in Fig. 1 C) due to their relatively long operational lifespan (half-life time c. 6 months [20]; maxima exceeding 3 years, [5] and personal observations), and the large size of the pitchers [21]. After feeding on pitcher prey or breakdown products, the dipterans leave the pitchers as adults, and consequently the plant will lose prey-derived nutrients [22], [23]. The only nutrients returned to the pitcher are excreta and larval/pupal exuviae. The emerging and dispersing adults will undoubtedly constitute a loss. So far, the magnitude of this kleptoparasitism in *N. bicalcarata* and other pitcher plant species remains unclear, but it is likely to be significant, as dipteran density is usually very high. Cresswell [24] found 74.7 larval and adult dipterans per pitcher of *N. bicalcarata* (sum of means of 10 dipteran taxa) and Clarke [25] reported means ranging from 27.6 to 114.6 (five sites, 9 dipteran taxa).

It has been speculated that the mechanical and metabolic activity of infauna and *C. schmitzi* ants might facilitate the digestive process of *Nepenthes* [9], [26]–[28], as has been suggested for the carnivorous American pitcher plant, *Sarracenia purpurea* [29]. However, investigations measuring nutrient uptake in *Sarracenia* found no effect of the presence of dipteran larval food webs [30], [31]. In a recent study on bromeliads, ^15^N-enriched leaf litter was fed to phytotelmata, but no difference was found in nitrogen uptake efficiency by the bromeliads between groups with and without detritivorous food webs, indicating that any facilitation of leaf litter breakdown by dipteran larvae was offset by their subsequent escape [32]. Most *Nepenthes* species are confronted with animal prey, which is generally easier to degrade than plant tissue. Thus, larval activity may be even less relevant for biomass breakdown than in bromeliads or *Sarracenia*. Also, for *N. bicalcarata*, such breakdown effects might be rendered irrelevant by the long operational lifespan of the pitchers.

Here, we investigate whether *C. schmitzi* ants increase the nitrogen supply for their host plant *N. bicalcarata* and by what mechanism. Using stable isotope analysis on natural and tracer-fed *N. bicalcarata* plants and by studying the interaction of *C. schmitzi* with the pitcher infauna, we address the following questions: (1) Is the net nutrient balance for *N. bicalcarata* improved when *C. schmitzi* is present? (2) Is there a flow of nutrients from *C. schmitzi* ants to the host plant? (3) Do *C. schmitzi* ants prey on dipteran infauna? (4) Do *C. schmitzi* ants reduce the number of infauna adults emerging?

## Results

### *C. schmitzi* Ants Enhance Nutrient Provision for *N. bicalcarata*

**Figure 2:**
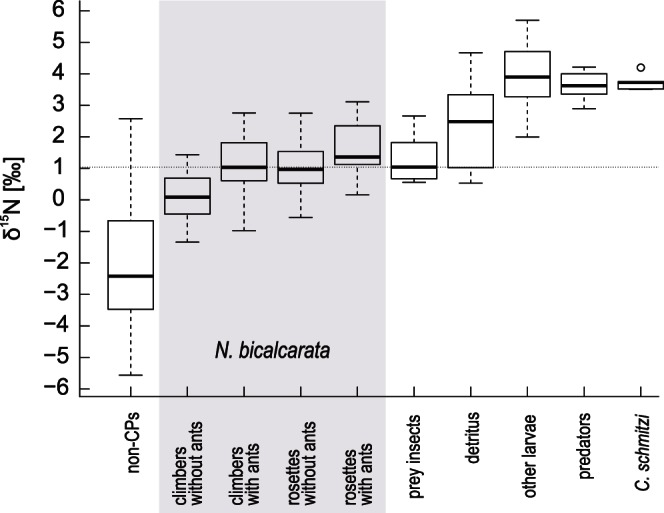
Natural δ^15^N for the components of the investigated food-web. Included are non-carnivorous plants (“non-CPs”, n = 7), *N. bicalcarata* plants (highlighted in grey; climbers without ants, n = 15; climbers with ants, n = 17; rosettes without ants, n = 15; rosettes with ants, n = 11), prey insects (n = 6), pitcher detritus (n = 10; one outlier with δ^15^N = 11.42 ‰ not shown), ‘other’ dipteran larvae (n = 10), ‘predatory’ dipteran larvae (n = 5) and *C. schmitzi* ants (n = 5). The dotted horizontal line highlights the level of prey insects. Boxplot shows medians, interquartile ranges, and the largest and smallest values that are not outliers (outliers shown as circles).

We found that the natural ^15^N/^14^N stable isotope abundance ratio (δ^15^N) of *N. bicalcarata* was significantly higher when colonised by *C. schmitzi* for both climbing and rosette stage plants, and that rosettes had a higher δ^15^N than climbers (Fig. 2; 2-way ANOVA; “growth habit” *F*_1,54_ = 8.13, *P* = 0.006; “ant colonisation” *F*_1,54_ = 10.53, *P* = 0.002; interaction of both factors *F*_1, 54_ = 1.42, *P* = 0.238). Two high outliers of δ^15^N were identified and were excluded from the analysis (one each from the colonised and uncolonised climbing plant groups).

We applied a two-end member mixing model (Equation 1) with mean values of δ^15^N_non-CP, n = 7_ = −1.95 ‰ and δ^15^N_insects, n = 6_ = 1.3 ‰ to estimate the relative contribution of insect-derived nitrogen in our 58 leaf samples (data of rosette and climbing plants pooled). The model suggests that colonised plants of *N. bicalcarata* received virtually all their nitrogen from prey insects (101.8±5.7%, n = 28; mean ± S.E.), whereas uncolonised plants received less from this source (77.6±5.5%, n = 30). Values >100% occurred when leaves had a higher δ^15^N than the prey insects, which may be based on isotope enrichment within the plant, as discussed by Schulze et al. [33].

The source of nitrogen did not appear to affect the concentration of nitrogen in foliar tissues (all groups: 1.20±0.018% of dry weight; mean ± S.E.), because neither *C. schmitzi* colonisation nor growth habit showed a significant effect (2-way ANOVA; “growth habit” *F*_1,54_ = 1.160, *P* = 0.286; “ant colonisation” *F*_1,54_ = 0.459, *P* = 0.501; interaction of both factors *F*_1,54_ = 0.150, *P* = 0.70).

### Trophic Relationships in the *N. bicalcarata* Phytotelm

As expected, food-web nodes (prey insects, detritus, other larvae, predatory larvae and *C. schmitzi*) differed significantly in their δ^15^N (Fig. 2; Kruskal-Wallis test; χ^2^_4_ = 14.755, n = 36, *P* = 0.0052). Post-hoc paired Wilcoxon-tests with Bonferroni correction revealed that prey insects were less enriched than *C. schmitzi* (*P* = 0.043), other larvae (*P* = 0.010) and predators (*P* = 0.043). The consumers were all very similarly enriched (*C. schmitzi* vs. other larvae, *C. schmitzi* vs. predators, predators vs. other larvae all *P*>0.2), while detritus held an intermediate position, differing from neither prey insects nor from *C. schmitzi*, other larvae or predators (all *P*>0.2).

### *N. bicalcarata* Absorbs Nitrogen from *C. schmitzi* Colonies

**Figure 3:**
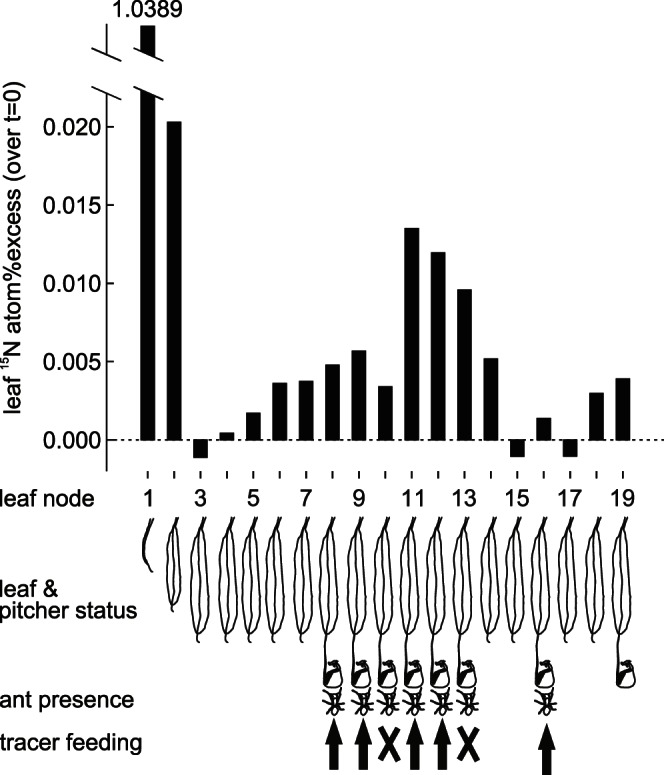
Flux of nitrogen from ant colony to pitcher plant. Bars indicate the change in ^15^N abundance in the leaves of a *N. bicalcarata* plant two weeks after feeding a ^15^N pulse to the symbiotic *C. schmitzi* colony. Leaf node 1 bears the youngest leaf. The pictogrammes under the graph explain the structure of the host plant, its ant colony and mark where tracer was fed.

Two weeks after feeding a pulse of ^15^N to a *C. schmitzi* colony, the ^15^N concentration was strongly increased in both host plant leaves (Fig. 3) and in the ants. The measured ^15^N atom% excess values (APE) exceeded natural fluctuations (plants: 0.002, ants: 0.001) by several orders of magnitude, demonstrating the arrival of our tracer. The strongest increase in ^15^N abundance occurred in the two youngest leaves. This identifies the developing leaves as the strongest above-ground sink for nitrogen. Leaves 3, 15 and 17 even lost ^15^N, and thus were net-source organs, whereas all others were net-sinks for the tracer.

The ants were considered as seven subcolonies for analysis, corresponding to the pitchers from which they were collected two weeks after the pulse. ^15^N tracer was clearly detected in the ant subcolonies from pitchers in which tracer had been fed (mean APE = 6.511±1.085, n = 5) and from those pitchers in which it had not (APE = 1.019 and APE = 10.452, n = 2), implying migration of workers between pitchers/transfer of food between workers (trophallaxis). Frequent exchange within the colony is further indicated by the lack of correlations between leaf APE and subcolony APE as well as between leaf APE and subcolony dry weight (Pearson’s correlation, only ant-colonised leaves included, both *P*>0.8).

We found that *N. bicalcarata* readily absorbed ^15^N-Glycine or its derivates directly from the pitcher fluid (APE for youngest leaf: 2.289). However, a strong increase in ^15^N abundance was also found following tracer injection into uncolonised domatia (APE for youngest leaf: 2.237). To clarify whether *C. schmitzi* transferred nitrogen via plant surfaces other than the pitchers, we fed tracer to the ants of a plant whose pitchers had been severed while the domatia and leaf bases remained intact. The ant colony was strongly enriched two weeks later despite not entirely consuming the offered tracer (APE = 1.327). Remarkably, tracer was also detected in this plant (APE for second youngest leaf: 0.014), confirming that *C. schmitzi* can also transfer nitrogen via other plant tissues.

### *C. schmitzi* Ants Prey on Dipteran Infauna

We trapped the emerging dipterans from 40 natural larval communities in *N. bicalcarata* lower pitchers. The overall number of emerged insects (ignoring taxonomy) was 160, corresponding to a rate of approximately four animals per pitcher per week. Both the Culicidae and the relatively small Chironomidae emerged in similar numbers unaffected by *C. schmitzi* colonisation (Table 1; χ^2^ goodness of fit test; both *P*>0.2). However, the larger Phoridae (the only family of Brachycera (true flies) in Table 1, all others being Nematocera) emerged almost three times more frequently from pitchers without *C. schmitzi* (Table 1; χ^2^ goodness of fit test; χ^2^_1_ = 6.368, *P* = 0.012). Other rare families identified were Corethrellidae (*Corethrella* sp.), Ceratopogonidae (*Dasyhelea* sp.) and Cecidomyiidae (*Lestodiplosis* sp.).

**Table 1:** Adult infauna emerged from *N. bicalcarata* pitchers. Unless otherwise stated, values are total numbers of individuals.

	ants absent (n = 20)	ants present (n = 20)
**Experimental conditions and plant characteristics:**		
mean sampling interval [d]	7.85	7.75
mean leaf node	6.3	6.3
peristome diameter ± S.E. [mm]	46.9±1.23	48.58±0.94
fluid volume ± S.E. [ml]	75.7±8.3	88.25±7.7
**Adult infauna emerged:**		
Chironomidae (*Polypedilum* spp.)	50	60
Phoridae	15	4
Culicidae	6	6
Ceratopogonidae (*Dasyhelea* sp.)	1	0
Corethrellidae (*Corethrella* sp.)	1	1
Cecidomyiidae (*Lestodiplosis* sp.)	0	1
undetermined specimens	8	7
** all taxa**	**81**	**79**

Brachyceran puparia were significantly more abundant when *C. schmitzi* was absent (Wilcoxon rank sum test on No. of puparia per pitcher; 60 ant-free pitchers, 67 ant-colonised pitchers, *W* = 3335.5, *P* = 4.38*10^−14^). Puparium counts in ant-free pitchers ranged from 0–12 with a median of one, and they contained a total of 130 puparia. In contrast, the ant-colonised pitchers held a total of only three puparia with a maximum of one and a median of zero.

**Figure 4:**
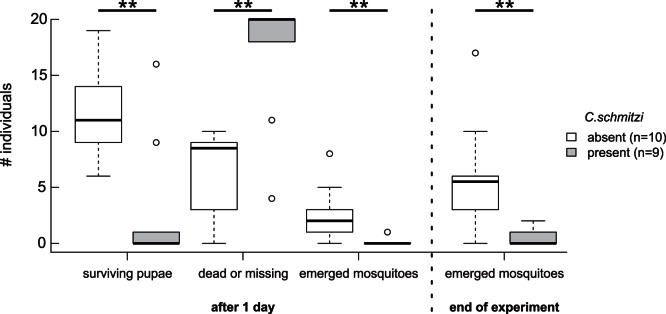
Effect of *C.schmitzi* presence on numbers of surviving *Aedes* sp. pupae and successfully emerging mosquitoes. The experiment started with 20 living pupae in each pitcher. Boxplot shows medians, interquartile ranges, and the largest and smallest values that are not outliers (outliers shown as circles).

We tested the behaviour of *C. schmitzi* towards aquatic mosquito pupae in *N. bicalcarata* pitchers. Within one day, ant presence significantly reduced the number of surviving pupae (Wilcoxon rank sum test; *W* = 78, *P* = 0.007) and the number of successfully emerging mosquitoes (Wilcoxon rank sum test; *W* = 78.5, *P* = 0.003), while the number of dead or missing individuals increased (Fig. 4; Wilcoxon rank sum test; *W* = 6.5, *P* = 0.002). Summing up the emerged adults over four days, after which no living pupae were left, significantly fewer mosquitoes emerged from pitchers containing *C. schmitzi* (Fig. 4; Wilcoxon rank sum test; *W* = 78, *P* = 0.006). A similar result was obtained for syrphids, where the number of surviving larvae after two days in a pitcher was strongly reduced by *C. schmitzi* (ants absent: 18 survivors out of 31; ants present: 1 survivor out of 30; Fisher’s exact test; *P* = 3.05*10^−6^).

Data from two pitchers with naturally occurring culicid infauna confirm predation by *C. schmitzi* ants (9 mosquitoes emerged from 11 pupae in one ant-free pitcher; 2 mosquitoes emerged from 11 pupae in one colonised pitcher; Fisher’s exact test; *P* = 0.009).

In the field, *C. schmitzi* also behaved aggressively towards experimentally offered mosquito pupae (Video S1 in Supporting Information). The ants captured them either from the fluid margin whilst standing on the pitcher wall or from within the fluid whilst swimming with the mandibles spread wide open. Ants grabbed pupae most frequently with their mandibles but when submerged, they occasionally also used their legs. At the fluid margin/pitcher wall, pupae were quickly pulled through the meniscus and dragged up the pitcher wall (the ant moving backwards). However, when they had caught a pupa in the fluid, the ants struggled to reach the pitcher wall because of the strong swimming movements of the pupa. Such a struggle could last for several minutes. While dragging a pupa through the fluid or along the fluid surface, the ants were observed to swim backwards, apparently using their middle and hind legs to generate backward thrust (in contrast to forward swimming, where the thrust is mainly produced by the front legs [12]). We are not aware of any other recording of backward swimming in insects; for the ants, swimming in reverse is apparently the only way they can approach the pitcher wall, as their front legs are blocked by the struggling prey. The reversing ants, adhering to the pitcher wall mainly by middle and hind legs, then pulled the pupae from the fluid. This behaviour was also observed with naturally occurring prey. Occasionally, ants that had already caught a pupa were joined by a second worker that also grabbed the struggling pupa.

Syrphid larvae were also observed under attack by swimming ants (Video S2 in Supporting Information). Biting made the maggots writhe but *C. schmitzi* were very persistent and only sometimes let go because of oxygen depletion (such "suffocated" ants drifted to the water surface where they regained mobility within a few seconds). The ants' attacks usually resulted in the maggot’s death and subsequent transport to their shelter underneath the peristome within minutes to hours.

## Discussion

Our results show that the presence of *C. schmitzi* ants increases the natural ^15^N/^14^N stable isotope abundance ratio (δ^15^N) in *N. bicalcarata* plants. Recently, a similar pattern was reported for *N. bicalcarata* plants of mixed growth phase, where it was shown that plants with many leaves, large total leaf area, low nutrient stress and more frequent pitcher production were more often occupied by *C. schmitzi* [17]. Higher δ^15^N and plant fitness were attributed to an increased prey capture rate caused by the “ambush behaviour” of *C. schmitzi* ants and to myrmecotrophy, i.e. the feeding of *N. bicalcarata* by its ant partner. However, nitrogen transfer from the ants to their host was not demonstrated, and it remained unclear whether the higher plant fitness was cause or consequence of ant occupation [17]. Our study demonstrates that *N. bicalcarata* plants are indeed fed by *C. schmitzi* ants. However, the higher δ^15^N of colonised plants cannot be explained by myrmecotrophy alone if the ants feed only on pitcher prey and nectar, because the cumulative waste of *C. schmitzi* colonies (carcases, excreta etc.) would not be enriched in ^15^N relative to the diet. Trophic enrichment occurs via the selective retention of ^15^N in body tissue, so the excreta of an animal are depleted in ^15^N relative to its diet [34], cancelling out any possible enrichment via the carcases of *C. schmitzi*. The isotope mixing model used by Bazile et al. [17] considers the nutrient input by *C. schmitzi* as a separate, external source of nitrogen and does not include nitrogen from soil or insect prey, leading to an overestimation of the nitrogen provision by myrmecotrophy.

Considering these factors, the elevated δ^15^N in colonised *N. bicalcarata* must reflect a change in the relative contributions of soil and prey nitrogen. *Nepenthes* have functional roots and may acquire substantial proportions of their nitrogen from the soil [27], [33]–[35]. Foliar δ^15^N, as well as total available nitrogen, are predicted to rise if more prey is captured. We applied an isotope mixing model that includes soil nitrogen and assumes *C. schmitzi* waste (i.e. excreta and carcases) to be homogenous with prey nitrogen. It appears that colonised *N. bicalcarata* obtain almost all of their nitrogen from insect prey and very little from the soil (for a discussion of values >100% see below), whereas uncolonised plants received only around 77% from prey. The lower value is in keeping with previous findings from other insectivorous *Nepenthes* species (61.5% in *N. mirabilis* [33]; 68.1% in *N. rafflesiana* [35]) that do not form symbioses with ants.

The reasons for the higher δ^ 15^N of *N. bicalcarata* rosettes compared to climbers are still unclear, but it could result from subtle differences in the prey spectra of lower and upper pitchers. Such differences have been reported for other *Nepenthes* species [36], [37]. For example, it has been shown for *N. rafflesiana* that lower pitchers capture relatively more ants than upper pitchers [36]. If a similar difference occurred in *N. bicalcarata*, it could potentially explain the observed higher δ^15^N of rosette plants.

Surprisingly, some *N. bicalcarata* had δ^15^N values exceeding those of prey insects, leading (with Equation 1) to ‘impossible’ values of more than 100% insect-derived nitrogen. It is unlikely that we underestimated δ^15^N_insects_, as the ants we sampled were typical representatives of insect prey and their δ^15^N varied only little. Levels of ^15^N in younger leaves exceeding those of prey insects have also been observed in other pitcher plant species by Schulze et al. [33]. These authors suggested that a plant-internal discrimination of ^15^N may occur, which would explain the impossible values of ≥100% insect nitrogen we obtained. In general, remobilisation and reallocation of nitrogen may cause intra-plant variation in δ^15^N [38]. If the involved physiological processes differ for climbers and rosettes, plant-internal discrimination could also contribute to the higher δ^15^N of rosette plants.

Regardless of these limitations, only ant colonisation can explain the observed difference in δ^15^N between colonised and uncolonised plants. Both plant-internal isotope discrimination and prey spectra are probably independent of *C. schmitzi* (and even if prey spectra differ, the prey δ^15^N values are likely to be comparable).

How is the origin of nitrogen related to available quantity? Soil nitrogen should have a similar availability to all *N. bicalcarata* in the same habitat, unaffected by *C. schmitzi*. There was no indication of local differences in soil nutrient availability in our field site, since vegetation was more or less uniform. Furthermore, all plant categories were sampled intermixed along transects. The high δ^15^N of colonised plants indicates that soil nitrogen is strongly diluted and masked by an overwhelming amount of insect nitrogen. In a habitat where nitrogen is limiting, plant fitness may be a function of the amount of insect-derived nitrogen. Thornham et al. [5] showed that pitchers of comparable size and age contained on average 1.45 times more prey ants when *C. schmitzi* were present. This difference is most likely a consequence of ant occupation rather than a cause, since the host cleaning behaviour of *C. schmitzi* enhances the capture rate of pitcher traps [5]. A positive effect on prey retention by the “ambush behaviour” of *C. schmitzi* has also been reported [4], but this effect could not be reproduced in our experiments [5].

We present evidence that the increased provision with insect-derived nitrogen also occurs through the ants' predation on infauna. *C. schmitzi* ants reduced the emergence rate of flies from *N. bicalcarata* pitchers, and they were effective predators of culicid pupae and syrphid larvae. Our ^15^N pulse-chase experiment confirmed that nitrogen ingested by *C. schmitzi* ants is taken up by *N. bicalcarata* plants. This demonstrates that the ants' predation on dipteran infauna will return nitrogen to the host plant which would have been lost otherwise. Exactly how much nitrogen is lost via emerging dipterans is still unknown, but we can make a rough estimate from our emergence trap data, where 81 Dipterans emerged from 20 pitchers in 7.85 observation days (i.e. 0.5 emerged insects per pitcher and day). Assuming a mean dry weight of 0.5 mg and 11% nitrogen [39] for each emerging fly, this would represent a nitrogen loss of 0.028 mg per day and pitcher. Shoots of *N. bicalcarata* have a growth rate of 31.9 days per leaf [5] and thus spend ca. 0.75 mg nitrogen per day on new leaves (calculated from a mean leaf dry mass of 4 g with 0.6% nitrogen [40]). As *N. bicalcarata* shoots typically have c. 5 active pitchers (median of 27 shoots), the loss by emerging infauna for the whole shoot amounts to ca. 0.14 mg per day or approximately 18.7% of the daily nitrogen consumption for growing new leaves. This suggests that plants would have access to 18.7% more nitrogen if the infauna did not escape. Thus, it is likely that even a moderate reduction of this loss by *C. schmitzi* ants will be of significant benefit to the plant.

Although previous authors have reported that *C. schmitzi* ants can catch mosquito larvae in the pitcher fluid [8], [16], the ants' predatory behaviour had not been observed in detail. Our video recordings confirm that *C. schmitzi* shows a vigorous hunting behaviour which is highly effective against active culicid pupae.

Our results suggest the ants' predation on infauna is selective, and clearly they do not eliminate dipteran kleptoparasitism entirely. In our emergence traps, the numbers of phorids were reduced, and fly puparia were virtually absent from pitchers colonised by *C. schmitzi*. True flies (Brachycera) are among the largest animals in pitcher communities, and their exclusion from ant-colonised pitchers may significantly reduce nitrogen losses. Considering that ant-colonised *N. bicalcarata* pitchers have a higher prey capture rate than uncolonised ones [5], the null hypothesis should be that they also support a higher infaunal biomass. Thus, the observed overall parity between ant-colonised and uncolonised pitchers in emergence frequency might indicate an interference of the ants with other fly families, too.

It is likely that *C. schmitzi* ants prey more efficiently on vulnerable stages (pupae) of dipterans than on their more mobile larvae. Clarke and Kitching [8] introduced 10 culicid larvae into ant-colonised and uncolonised pitchers and found a small but significant decrease in larval survival attributable to the ants after one week (9.6 survived in uncolonised pitchers compared to 7.6 in colonised pitchers). Compared to the effect on culicid larvae, the ants' effect on culicid pupae observed in this study was much stronger.

Our analysis of trophic relationships in the *N. bicalcarata* pitcher food web indicates that all the “predators” examined, i.e. *C. schmitzi, Toxorhynchites* sp. and *Corethrella* sp., were actually feeding at a similar trophic level as their presumed food source, the other phytotelm larvae. This surprising result suggests that these insects were predominantly scavengers of drowned prey rather than hunters, in contrast to previous notions [19], [21], [22], [40]–[42]. Thus, the predation of infauna by *C. schmitzi* may be facultative or opportunistic [8]. However, it is possible that an existing difference in trophic level between *C. schmitzi, Toxorhynchites* sp., *Corethrella* sp. and the group of “other larvae” has been obscured, because the latter group included consumers of microscopic infauna (filter feeders) and potentially other predators. Similarly, the intermediate/overlapping δ^15^N of detritus between prey and macro-invertebrate consumers may result from colonisation of the particles with microscopic infauna (bacteria, nematodes, mites, etc.), which may comprise several trophic levels in itself.

Interestingly, foliar nitrogen concentration was not increased by partner ant colonisation, in agreement with the results of Bazile et al. [17]. Apparently, better nutrition enhances growth in *Nepenthes* but does not increase foliar nutrient concentration. Moran and Moran [14] found that prey-deprived *N. rafflesiana* plants had a less efficient photosynthesis and produced less biomass than a fed control group, but tissue nitrogen and phosphorus concentrations were not reduced. A higher biomass production in fed plants was also found in *N. talangensis* and *N. ampullaria* [43], [44]. As *N. bicalcarata* with *C. schmitzi* are likely to have a better supply of nutrients, their growth rates may be higher, making it beneficial for *N. bicalcarata* plants to house *C. schmitzi* ants. A further positive effect of *C. schmitzi* for *N. bicalcarata* is that the ants may disperse nutrients both in space (through movement between pitchers) and time (through temporary 'sequestration' in the ants’ bodies) on the host plant. *C. schmitzi* colonies may buffer fluctuations of nutrient availability such as mass capture events or capture of very large prey items [8] by feeding on such “excess” prey and then returning nutrients as waste more continuously.

*C. schmitzi* provides nutritional benefits to its host plant not only by increasing the rate of pitcher formation and enhancing the pitchers’ capture rate [4], [5], but also by preventing nutrient export via dipteran kleptoparasites, as shown in this study. Uniquely, feeding of *N. bicalcarata* by *C. schmitzi* ants does not occur by the active collection of nutrients from outside the host plant as in other ant-fed plants [45]–[48]. It is likely that the nutritional benefits more than compensate the costs that *N. bicalcarata* might incur for housing the ants, such as the possible nutrient export via winged males and queens of *C. schmitzi* itself, the production of hollow tendril domatia and an increased nectar secretion [18]. Our study adds to a growing body of evidence showing that many alternative nutrient acquisition strategies have evolved in the genus *Nepenthes* [27], [37], [49]–[54].

The ecological role of *C. schmitzi* is comparable to that documented for aquatic predators attacking dipteran kleptoparasites in bromeliad phytotelmata [32], [55]. Kleptoparasitism is known for many other carnivorous plants [56]–[58] and it is likely that selection pressure has favoured plant adaptations to combat the resulting nutrient loss. Carnivorous plants may have evolved direct defences against kleptoparasites such as actively closing traps which help to make prey less accessible to thieves (e.g. venus fly trap or sundew leaves [59]–[63]). The nutritional interaction between *N. bicalcarata*, pitcher infauna and *C. schmitzi* ants presented here constitutes a novel type of indirect plant defence against kleptoparasites, similar to the biotic defence by ants against herbivores and plant pathogens [64].

## Materials and Methods

### Field Work

Experiments were carried out in the Badas peat swamp forest (PSF) in Brunei Darussalam (4°33'34"N, 114°25'16"E). Leaf samples of *N. bicalcarata* were taken there and at three nearby sites along Labi Road, Belait district (one disturbed Alan Bunga PSF, one swampy kerangas secondary growth and one primary forest with intermixed patches of peat swamp and mixed dipterocarp). Our work was conducted under the research permit UBD/PSR/5(a) issued by the Brunei Department of Forestry and *Nepenthes* samples were exported to the UK for analysis under CITES permit BA/MAP/191/1108.

### Stable Isotope Natural Abundance

#### a) Effect of *C. schmitzi* and growth habit on *N. bicalcarata* isotope signature

To assess the effect of *C. schmitzi* ants on the nutrition of *N. bicalcarata*, we analysed the amount and stable isotope composition for nitrogen (δ^15^N) of plants with at least three pitchers that were either uncolonised or colonised by *C. schmitzi*. "Uncolonised" plants were free of *C. schmitzi* and showed either no traces of previous colonisation or their last deserted domatium entrance was at least five leaf nodes below the sampled leaf, indicating that the plant had been without *C. schmitzi* ants for at least three months [5]. “Uncolonised” plants were only included if all adjacent *N. bicalcarata* and those with a possible rhizome connection were also ant-free. Each sample was taken from a different plant, and study plants belonged to clearly separated stands to avoid sampling from the same plant clone. The youngest offshoots from rhizomes were excluded to ensure that the nitrogen sequestration of sampled shoots was as independent as possible. 2×2 cm samples were cut from the middle of the leaf belonging to the youngest fully functional ( = open) pitcher, as in previous studies [35], [37]. Samples were washed and dried at 80°C on the day of collection.

We also compared climbing plants (climbing stems with at least 1 m of length) with non-climbing rosettes (at least 80 cm in diameter, consisting of at least four fully developed leaves and some independent stem). Leaf samples had a comparable age within the climbing/rosette categories, since the leaf node from which the sample was taken was similar between the colonised and uncolonised plants (2-way ANOVA on “leaf node”; “growth habit” *F*_1,55_ = 19.76, *P*<0.001; “ant colonisation” *F*_1,55_ = 0.45, *P* = 0.5). Furthermore, the total stem length above ground was also similar between the colonised and uncolonised plants (2-way ANOVA on “total stem length”; “growth habit” *F*_1,43_ = 12.12, *P* = 0.001; “ant colonisation” *F*_1,43_ = 1.21, *P* = 0.28; these data were available for 46 of the study plants).

The δ^15^N of *N. bicalcarata* did not differ between the different study sites (Kruskal-Wallis tests, all *P*≥0.2).

#### b) Estimation of the amount of insect-derived nitrogen in *N. bicalcarata*

We estimated the contribution of insect-derived nitrogen as opposed to soil-derived nitrogen to the nutrition of *N. bicalcarata* using a two-end member mixing model [47], [65]:
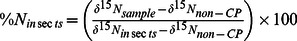
(1)where δ15Nnon-CP is the mean δ15N of seven species of sympatric, non-carnivorous reference plants, and δ15Ninsects is the mean δ15N of typical insect prey of *N. bicalcarata*. The δ15N of the non-carnivorous reference plants was used as an estimate of the δ15N of soil-derived nitrogen. Samples were taken as before from plants from the herb to tree strata at the Badas site. The species sampled were *Alocasia longiloba* (Araceae), *Clerodendrum fistulosum* (Lamiaceae), *Macaranga puncticulata*, *M. caladiifolia*, *M. bancana* (Euphorbiaceae), *Pandanus andersonii* (Pandanaceae) and *Shorea albida* (Dipterocarpaceae). δ15Ninsects was used to estimate the insect-derived nitrogen signature in *N. bicalcarata*, assuming that no isotopic fractionation takes place during digestion and absorption by the plant. As more than 90% of the prey animals of *N. bicalcarata* are ants [3], [4], we sampled six morphospecies of ants that are frequently found in the pitchers or foraging on *N. bicalcarata* (three *Camponotus* spp., two *Crematogaster* spp., one unidentified species; one worker each) to obtain δ15Ninsects. We avoided the sampling of drowned animal remains from pitchers to exclude a possible isotopic alteration by digestive processes and contamination by microfauna.

#### c) Assessment of trophic relationships in *N. bicalcarata* phytotelmata

Food web components were sampled from ten pitchers of *N. bicalcarata* (see Table 2). Entire *C. schmitzi* sub-colonies were harvested comprising all developmental stages from a pitcher and the appendent domatium. Aquatic larvae were killed by freezing and sorted under a stereo microscope into two categories “predators” (*Toxorhynchites* sp. and *Corethrella* sp.) and “other larvae” before drying as before. From each pitcher, several larvae were pooled as a single sample. Detritus samples included the empty exoskeletons of pitcher prey; we removed any discernible living infauna under the stereo microscope and dried the sample immediately. For “prey insects”, we used the same data from six morphospecies of ants as for the isotope mixing model (above).

**Table 2:** Samples taken for the analysis of the natural abundance of ^15^N in *N. bicalcarata* phytotelm food webs.

*C. schmitzi*	pitcher type	leaf node	samples (each item = one combusted sample)
yes	aerial	16	ant colony, other larvae, Corethrella, detritus
yes	aerial	6	ant colony, other larvae, detritus
yes	ground	3	ant colony, other larvae, Toxorhynchites, detritus
yes	aerial	14	ant colony, other larvae, Toxorhynchites, detritus
yes	aerial	6	ant colony, other larvae, detritus
no	aerial	10	other larvae, detritus
no	aerial	5	other larvae, detritus
no	ground	5	other larvae, Toxorhynchites, detritus
no	ground	3	other larvae, Toxorhynchites, detritus
no	aerial	10	other larvae, detritus

Each of the ten pitchers grew on a different plant, and food web components were sampled and analysed from each pitcher separately. “Other larvae” refers to Brachycera and putative non-predatory Nematocera.

### Flux of Nitrogen from Ant Colony to Host Plant: Pulse-chase Experiments

We tested whether *N. bicalcarata* can absorb the nitrogen contained in a *C. schmitzi* colony, and if so, which surfaces of the plant are capable of absorbing. Four treatments were performed, each on a different plant. We used ^15^N-glycine (99 atom% ^15^N, Sigma-Aldrich Company Ltd., Gillingham, UK) as a tracer. ^15^N-glycine was dissolved to give a ^15^N-concentration of 1.302 M (100 mg of 99 atom% ^15^N-glycine per ml) in either water (“water tracer”) or in a 1.25 M (30 mass-%) sucrose solution (“sugar tracer”). The solution was administered to the plants or to ant feeders using a microliter pipette. Samples for isotopic analysis were taken both immediately before tracer application (as control) and 14 days later. Leaves were sampled twice, from their tips, left of the midrib at t = 0 and from their middle, avoiding the midrib, at t = 14 d. In all except for the first treatment, only the youngest leaves were sampled. Two ants were sampled from each pitcher at t = 0. Whole colonies including workers, alates and brood from the domatia were collected separately for each pitcher at t = 14 d. Ants were first stored in alcohol (storage in alcohol has no isotopic effect [66]), then dried and ground to a powder to analyze ^15^N abundance for the whole colony or subcolonies as appropriate. Results were calculated as APE (atom% excess), defined here as the difference in atom% (percentage of ^15^N among all nitrogen isotopes) between the samples of t = 0 and t = 14 d.

The following experiments were conducted:

#### a) Nitrogen transfer from *C. schmitzi* to *N. bicalcarata*

50 µl sugar tracer were offered to the *C. schmitzi* colony in each of five pitchers on a *N. bicalcarata* plant of around 1.4 m diameter that had eight pitchers in total. Thus, a total of 250 µl tracer (n(^15^N) = 325.4 µmol; m(^15^N) = 4.882 mg) was fed to the ant colony. We mounted ant feeders inside *N. bicalcarata* pitchers in order to exclude visitors other than *C. schmitzi* and to avoid direct contact between tracer and host plant. The feeders consisted of lidless Eppendorf tubes, held upright, with a droplet of tracer solution inside. The depth of the feeders ensured that the ants would need to take several steps after drinking and thereby wipe off any tracer fluid that might stick to their feet. We observed that the animals avoided stepping into the fluid while drinking from it. A plastic bag was placed over the whole pitcher to exclude rain and flying insects. Ants were allowed to ingest the solution for two full days, after which no more liquid was left inside the feeders. After 14 days, all 19 leaves on the plant were sampled.

#### b) Absorptive surfaces of *N. bicalcarata*

To investigate which surfaces of *N. bicalcarata* that are frequently occupied by *C. schmitzi* have the potential to absorb nitrogenous compounds, we applied water tracer directly to two uncolonised rosette plants, each with a diameter of 60 cm. One plant was given 50 µl water tracer into each of its three pitchers, totalling 195.3 µmol of ^15^N-glycine. In the other plant, the unopened domatia in three pitcher tendrils were carefully cut open using a scalpel and into each, 50 µl of the same tracer solution were injected, ensuring that no tracer came into contact with the tendril wounds. The wounds were then sealed with Tanglefoot® (Contech Ent. Inc., Victoria, BC, Canada).

#### c) Nitrogen transfer from *C. schmitzi* to *N. bicalcarata* without pitchers

To examine whether *C. schmitzi* are able to transfer nitrogen to their host via surfaces other than the pitcher, we selected a plant of similar shape as in the first treatment, but with a smaller *C. schmitzi* colony (assessed by counting ants under the pitcher rim using a dentist’s mirror). In this treatment, all pitchers on the plant were cut from their tendrils at the point of insertion to interrupt vascular flow, and then mounted back onto the tendril to allow the ants to move between the pitcher and the domatia as under natural conditions. This procedure left the domatia intact, but it inevitably led to draining of the pitcher fluid. Tracer–sucrose solution was supplied in ant feeders in the same way as explained above in three of the four pitchers of the plant. The ants were allowed to feed on the tracer solution for seven days, after which some of it was still left.

### Stable Isotope Chemical Analysis

Dried samples were analysed for their nitrogen isotope composition using an elemental analyser (Costech International SpA, Milan, Italy) attached to a MAT 253 mass spectrometer (Thermo Fisher Scientific Inc., Waltham, USA). Weighed samples of standards were analysed at various points throughout the run allowing percentage nitrogen to be calculated for the batch of samples. Calibration reference standards for δ^15^N (air) were obtained from IAEA in Vienna, and were run in parallel with the samples. Precision of analyses was ±0.5% for amounts of nitrogen, and better than 0.1 ‰ for ^14^N/^15^N.

### Interaction of *C. schmitzi* with Aquatic Dipteran Larvae

#### a) Rate of dipteran emergence from natural pitcher communities

**Figure 5:**
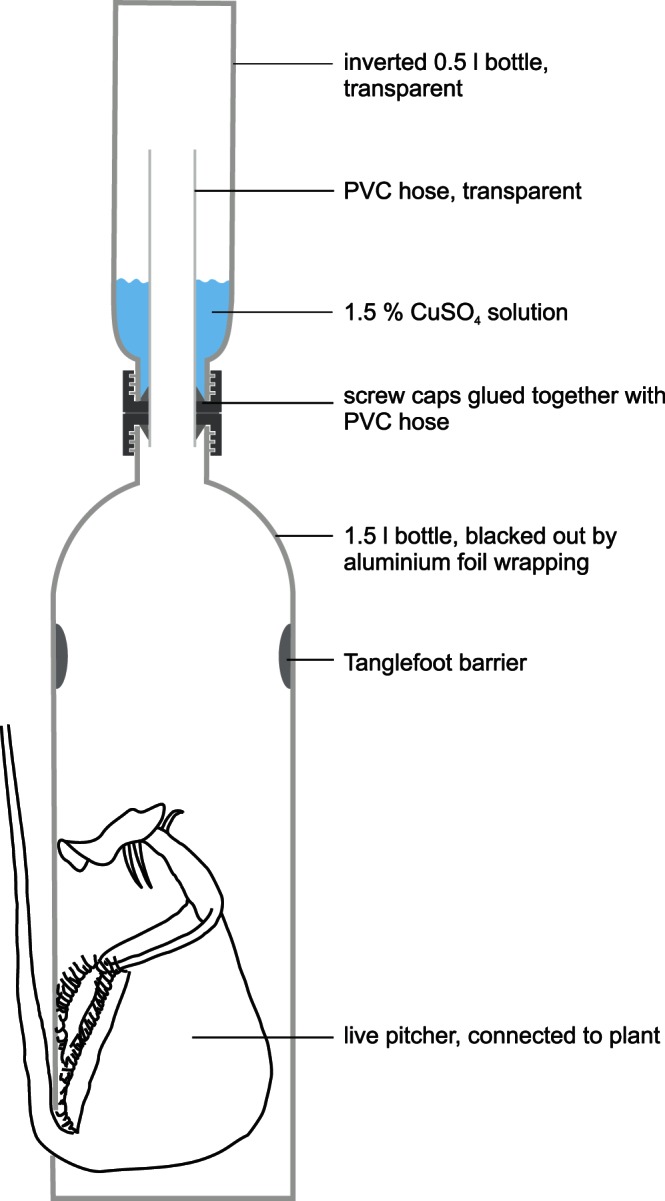
Schematic of “emergence trap” for pitcher plant infauna. The lower bottle containing the pitcher is completely darkened, causing emerging dipterans to move upwards towards the light shining through the neck. Tanglefoot® prevents crawling insects from entering the collection bottle. The fish trap-like design prevents escape from the collection bottle. Emerging insects reaching the collection bottle were killed when falling into a 1.5% CuSO_4_ solution. Traps were stabilised with a pole. Whole, living pitchers could be used without significant interference. Although *C. schmitzi* could freely pass through a small gap in the bottle along the pitcher tendril, the trap was mosquito-tight (without ants, all the 20 inserted individuals were always recovered; n = 10 traps).

To test whether *C. schmitzi* ants affect the numbers of dipterans emerging from pitchers, we equipped 20 pitchers from plants colonised by *C. schmitzi* and 20 pitchers from uncolonised plants with specially designed "emergence traps" (Fig. 5). The traps consisted of a sealed enclosure around the pitcher leading into a collection bottle containing 1.5% CuSO_4_ solution in which emerging insects were killed. From the collection bottle, insects were transferred to 70% ethanol, counted and identified to family level under a stereo microscope using identification keys [67], [68]. We ensured that pitchers in both groups had comparable ages (leaf node numbers), controlling for possible age effects on the infaunal community. Traps were set up for 7–9 days in June 2011, ensuring that sampling interval did not differ between the groups (Wilcoxon signed rank test; *W* = 210, *P* = 0.78). In the colonised pitchers, the number of *C. schmitzi* ants decreased slightly but not significantly over the trapping period (start: 12.9±3.14 ants; end: 11.6±3.20 ants (mean ± S.E.); paired Wilcoxon signed rank test; *V* = 143, *P* = 0.054). Habitat (pitcher) size tended to be larger in colonised pitchers but was also not significantly different between the treatment groups (peristome diameter: Wilcoxon rank sum test; *W* = 139, *P* = 0.15; fluid volume: Wilcoxon rank sum test; *W* = 135.5, *P* = 0.083).

#### b) Abundance of fly puparia in pitchers

We investigated whether *C. schmitzi* presence has an effect on the abundance of infauna puparia of (brachyceran) fly species that pupate on the inner wall of *N. bicalcarata*. Between 4 June 2011 and 13 July 2011, empty and living puparia were counted in randomly chosen ant-colonised (n = 67) and uncolonised pitchers (n = 60) using a dentist’s mirror. Additionally, approximately 400 pitchers of *N. bicalcarata* were visually examined for Brachycera larvae presence in the fluid. When found, notes were taken on the colonisation status of the pitcher by *C. schmitzi* ants. Ten larvae and two puparia were collected and reared in plastic cups for identification. Although most larvae began pupation, only two were successfully reared. One of these was *Wilhelmina nepenthicola*, the other was *Nepenthosyrphus oudemansi* [69]. A third species was putatively identified as *Nepenthomyia* sp. from larval characters.

#### c) Test of the predatory behaviour of *C. schmitzi* against aquatic Diptera

To test the hypothesis that *C. schmitzi* predates pitcher infauna, we observed the ants’ interactions with syrphid larvae (*Eristalis* sp.) and culicid pupae (*Aedes* sp.) released into the pitcher fluid.

As previous studies had reported relatively low predation rates for culicid larvae [8], and larvae of Culicidae, Chironomidae and Ceratopogonidae are commonly found in ant-colonised pitchers, we used pupae as one of the culicids' most vulnerable life stages. We tested the effect of *C. schmitzi* on the survival and emergence of culicid pupae collected from a drainage ditch where they were available in very large numbers. Individual pitchers, one per plant, were emptied of their contents using a modified aspirator (pooter). The fluid was then replaced with tap water to facilitate identification later. After counting the number of *C. schmitzi* ants under the peristome, 20 healthy mosquito pupae were released into the pitcher fluid. The pitcher was then enclosed in a transparent 1.5 l plastic bottle (Fig. 5, but without upper collection bottle), which allowed free passage for ants but trapped emerging flies. The experiment was repeated for nine *C. schmitzi* colonised plants and ten uncolonised plants. Bottles and pitcher fluids were inspected daily, until no more living mosquito pupae could be found (they had either died, emerged or disappeared). The number of surviving mosquito pupae, dead or missing individuals as well as living, emerged adults after one day was counted and compared between ant-colonised and control pitchers, as was the total (final) number of successfully emerged adults.

On one occasion, we were able to collect 22 culicid pupae from a single *N. bicalcarata* pitcher, belonging to two morphospecies. This allowed us to repeat the above experiment with natural infauna. Eleven pupae each (9+2 specimens from each morphospecies per pitcher) were put into one ant-colonised and one ant-free control pitcher. As before, pitchers were enclosed in emergence traps and inspected daily, collecting the same data as before.

A variation of the above experiment was performed using third instar syrphid larvae, again collected from a drainage ditch in large numbers. Here, one syrphid larva was put into each of 30 *N. bicalcarata* pitchers with *C. schmitzi* present. As few uninhabited pitchers were available and as the larval mortality was assumed to be independent of the number of individuals in one pitcher 31 syrphid larvae were put into 12 uncolonised pitchers with a maximum of three larvae per pitcher. The pitchers were enclosed in emergence traps and the number of surviving syrphid larvae after two days was compared between ant-colonised and control pitchers.

The behaviour of *C. schmitzi* towards the 'model' culicid pupae and syrphid larvae was documented on video (Sony Handycam DCR-SR35E, Sony Corp., Japan).

## Supporting Information

Video S1 **Movie of** ***Camponotus schmitzi*** **ants showing aquatic hunting for mosquito pupae.**
(AVI)

Video S2 **Movie of a** ***Camponotus schmitzi*** **ant showing aquatic hunting for a large fly larva.**
(AVI)
